# Modeling Mucopolysaccharidosis Type II in the Fruit Fly by Using the RNA Interference Approach

**DOI:** 10.3390/life10110263

**Published:** 2020-10-30

**Authors:** Laura Rigon, Nicole Kucharowski, Franka Eckardt, Reinhard Bauer

**Affiliations:** 1 Molecular Developmental Biology Unit, Life & Medical Sciences Institute (LIMES), University of Bonn, Carl-Troll-Straße 31, 53115 Bonn, Germany; s0nikuch@uni-bonn.de (N.K.); f.eckardt@uni-bonn.de (F.E.); 2 Fondazione Istituto di Ricerca Pediatrica “Città della Speranza”, Corso Stati Uniti 4, 35127 Padova, Italy; 3 Neuronal Wiring, Life & Medical Sciences Institute (LIMES), University of Bonn, Carl-Troll-Straße 31, 53115 Bonn, Germany

**Keywords:** Mucopolysaccharidosis, lysosomal storage disorders, Hunter Syndrome, *Drosophila melanogaster*, fruit fly, RNA interference

## Abstract

Mucopolysaccharidosis type II (MPS II) is a lysosomal storage disorder that occurs due to the deficit of the lysosomal enzyme iduronate 2-sulfatase (IDS) that leads to the storage of the glycosaminoglycan heparan- and dermatan-sulfate in all organs and tissues. It is characterized by important clinical features and the severe form presents with a heavy neurological involvement. However, almost nothing is known about the neuropathogenesis of MPS II. To address this issue, we developed a ubiquitous, neuronal, and glial-specific knockdown model in *Drosophila melanogaster* by using the RNA interference (RNAi) approach. Knockdown of the *Ids/CG12014* gene resulted in a significant reduction of the *Ids* gene expression and enzymatic activity. However, glycosaminoglycan storage, survival, molecular markers (*Atg8a, Lamp1, Rab11*), and locomotion behavior were not affected. Even strongly reduced, IDS-activity was enough to prevent a pathological phenotype in a MPS II RNAi fruit fly. Thus, a *Drosophila* MPS II model requires complete abolishment of the enzymatic activity.

## 1. Introduction

Mucopolysaccharidosis type II (MPS II, or Hunter Syndrome, MIM #309900) is a rare X-linked lysosomal storage disorder caused by the deficit of the iduronate 2-sulfatase (IDS, EC3.1.6.13) enzyme, leading to storage of the glycosaminoglycans (GAGs), heparan- and dermatan-sulfate in all organs and tissues. MPS II has an incidence rate of 0.38–1.09 per 100,000 live newborns [1]. It is characterized by progressive important clinical features, including organomegaly, cardiopathies, joint stiffness, multiple dysostoses, and skeletal deformities [2]. Patients are usually classified in attenuated or severe based on the absence or presence of the neurological involvement. The attenuated form presents normal intelligence and extended life expectancy. Patients with severe phenotype are characterized by a heavy neurological involvement already present in infancy, with impaired cognitive abilities, delayed mental development, and behavioral problems. No differences in the IDS enzymatic activity were observed between the attenuated and severe form, and all MPS II patients present no residual IDS activity [2]. The severe form occurs in about 2/3 of the patients and death occurs in the first-second decade of life [3]. However, very few studies have been conducted on the neuropathogenesis of this disease [4,5,6,7,8], and the currently available enzyme replacement therapy clears GAGs from the peripheral tissues, but not from the central nervous system, as the recombinant enzyme does not cross the blood–brain barrier. Therefore, comprehension of the brain pathogenesis is essential for the identification of innovative, effective therapeutic targets.

Nowadays, mice are the animal model of choice for in vivo studies in mucopolysaccharidoses. However, in recent years, simpler models have been used for basic and therapeutic studies providing excellent results. Two *Danio rerio* (zebrafish) models have been developed for MPS II, highlighting important findings concerning the skeletal and cardiac pathology [9,10,11]. Recently, also two *Drosophila melanogaster* (fruit fly) models have been reported for MPS IIIA and MPS VII [12,13]. Even if realized with two different approaches (knockdown for MPS IIIA and knockout for MPS VII), both fruit fly models reflect well the human disease characterized by reduced MPS IIIA and MPS VI enzymatic activity, shortened life span, elevated GAGs and acidification of lysosomes, as well as reduced climbing ability. Moreover, in the MPS IIIA fly model it was shown that genes regulating oxidative stress and autophagy contribute to the neuropathology of the disease [12]. In the MPS VII knockout, it was found that extensive apoptosis leads to muscle degeneration and loss of dopaminergic neurons with a final locomotor deficit [13].

*Drosophila melanogaster* has been used for genetic studies for more than a century. Short generation time, the ease of maintenance, and the genetic and molecular tools available to study flies make the fly a wonderful genetic system to do gene manipulation and pathway analysis [14]. Moreover, the degree of functional conservation between *Drosophila* and mammalian genes is high. About 75% of human disease genes have a functional homolog in the fruit fly [15]. In addition, the organ systems are functionally highly conserved from *Drosophila* to mammals, sharing evolutionarily conserved molecular mechanisms that underlie cellular and physiological processes [14]. All this has allowed many insights in neuronal development and the modeling and studying of different neurodegenerative disorders, like Alzheimer’s Disease, Parkinson’s Disease, Huntington’s Disease, Amyotrophic Lateral Sclerosis, and Ataxia Telangiectasia [16,17]. 

The *Ids* gene also has a single homologue/ortholog in *D. melanogaster* (*Ids/CG12014*), which is highly expressed in the central nervous system [18]. The amino acid sequence of human IDS shares 47% identity with the one of the fruit fly [18]. Therefore, we set out to develop a fruit fly model for MPS II with the main objective of having a simpler model, especially to study the early stages of neuronal development as the severe phenotype occurs as early as infancy. In this study we used the RNA interference (RNAi) approach to knockdown the *Ids* gene both ubiquitously and specifically in neurons and glial cells. Here, we report the data, which we obtained from the MPS II RNAi fruit fly model. 

## 2. Materials and Methods 

### 2.1. Fly Stocks and Husbandry

The following fly stocks were obtained from the Bloomington Drosophila Stock Centre (Dept. Biology, Indiana University, Bloomington, IN, USA): *w^1118^* (#5905); P{TRiP.HMC03475}attP40 (dsRNA for RNAi of *Ids*, #51901); P{TRiP.HMJ30081}attP40 (dsRNA for RNAi of *Ids*, #63004); P{Act5C-GAL4}25FO1 (*act*-Gal4, #4414); P{GAL4-elav.L}3 (*elav*-Gal4, #8760); P{GAL4-repo} (*repo*-Gal4, #7415). A third line of dsRNA for RNAi of *Ids* was obtained from the Vienna Drosophila Resource Center (#13990). For each *Ids*-RNAi line (BL51901, BL63004, v13990), crosses with *act*-Gal4, *elav*-Gal4, and *repo*-Gal4 were established. Virgins of the Gal4 lines were crossed either with males of the *Ids*-RNAi lines or with males of *w^1118^* as a negative control. All flies were maintained on a fortified medium composed of 1% agar, 1% glucose, 6% fresh yeast, 9.3% molasses, 8.4% coarse semolina, 0.9% acid mix, and 1.7% tegosept. All crosses were carried out at 29 °C and flies maintained at 25 °C.

### 2.2. Real-Time qPCR

Tissues were homogenized using a Precellys 24 homogenizer (Bertin Technologies, Darmstadt, Germany) and RNA was isolated from 1-day-old adult flies using TRIzol™ Reagent (Thermo Fisher Scientific, Darmstadt, Germany) according to the manufacturer’s protocol. When the RNAi lines were ubiquitously expressed in combination with the *act*-Gal4 driver line (*act*-Gal4 progeny), 5 flies were homogenized for each experiment. When the RNAi lines were expressed in neurons or glial cells in combination with the *elav*-Gal4 or *repo*-Gal4 driver lines (*elav*-Gal4 or *repo*-Gal4 progeny), 15 heads were homogenized for each experiment. cDNA was generated using the LunaScript™ RT SuperMix Kit (New England BioLabs, Frankfurt am Main, Germany). Real-Time PCR (RT-qPCR) was performed using Luna^®^ Universal qPCR Master Mix (New England BioLabs) with a CFX Connect Real-Time PCR Detection System (Bio-Rad, Feldkirchen, Germany). Values were normalized against Ribosomal protein 49 (rp49) as reference gene and wild type control (ΔΔCq). Table 1 shows the sequences of the gene specific primers used. Each experiment was repeated in triplicate at least 5 times.

### 2.3. IDS Activity Assay

One-day-old adult flies were homogenized in 0.9% NaCl added with protease inhibitors (cOmplete™ ULTRA Tablets, Mini, EDTA-free, EASYpack Protease Inhibitor Cocktail, Roche), using a Precellys 24 homogenizer (Bertin Technologies). Samples were centrifuged for 5 min at 1000 rcf at 4 °C to eliminate debris, the supernatants were recovered, and protein concentration was determined by using the Bradford protein assay. IDS activity was evaluated by a fluorometric assay employing the substrate 4-methylumbelliferyl a-L-idopyranosiduronic acid 2-sulfate disodium salt (Biosynth Carbosynth) [19]. Briefly, 20 µg of protein per sample were analyzed adding 20 µL of 1.25 mM substrate and incubating at 37 °C for 4 h. 20 µL of McIlvaine buffer pH 4.5 and 30 µL of alpha-L-iduronidase enzyme (Aldurzyme^®^, Biomarin, Novato, CA, USA) were added to each sample and incubated at 37 °C for further 20 h. The reaction was stopped adding 1420 µL of stop buffer (0.5 M NaHCO3/0.5 M Na2CO3 pH 10.7, 0.025% Triton X-100). Fluorescence was measured at 355/460 nm excitation/emission in an Infinite^®^ 200 PRO microplate reader (Tecan, Männedorf, Switzerland). 4-Methylumbelliferone (Sigma-Aldrich, Darmstadt, Germany) was used as standard. For each experiment, 5 flies were used for *act*-Gal4 progeny, while 15 heads were homogenized for *elav*-Gal4 and *repo*-Gal4 progeny. Each experiment was repeated at least 5 times.

### 2.4. GAG Assay

One-day-old adult flies were homogenized using a Precellys 24 homogenizer (Bertin Technologies) in 0.9% NaCl + 0.2% Triton X-100, left under stirring overnight at 4 °C and centrifuged for 5 min at 1000 rcf at 4 °C to eliminate debris. Supernatants were recovered and protein concentration was determined using the Bradford protein assay. GAG content was measured by using Björnsson’s protocol [20] with modifications, as previously described [21]. For each experiment, 15 whole flies were used for *act*-Gal4 progeny, while 45 heads were homogenized for *elav*-Gal4 and *repo*-Gal4 progeny. Each experiment was repeated at least 4 times.

### 2.5. Lethality Assays

First instar (L1) larvae were collected on apple juice agar plates (2% agar, 2.5% sucrose, 25% apple juice, 1.5% nipagin) (20–25 larvae per plate) and supplied with fresh yeast paste (42 g of yeast mixed with 9 mL of tap water). The number of emerging pupae and adults (alive 24 h after hatching) was counted. At least 100 larvae were analyzed for each genotype.

### 2.6. Crawling Assay

To analyze the larval locomotion a crawling assay was performed. Five L3 larvae at a time were transferred onto a 2% PBS-agarose plate (10-mm petri dish), at room temperature. The plate was put in a box with controlled light intensity and a graph paper, as a reference measure, and left for 1 min to acclimate. Then, they were filmed for 1 min using a smartphone and their movement was tracked for 30 s with ImageJ (Fiji) as previously described [22]. Ninety to one-hundred larvae were analyzed for each genotype. 

### 2.7. Climbing Assay

The climbing assay was performed as previously described [13] with small modifications. Briefly, for each group 15–20 flies were collected and let recover for 24 h (h) to avoid effects due to anesthesia. Then, they were flipped into a glass tube (19.5 cm × 2.7 cm), gently tapped to the bottom and the number of flies that reached the 10 cm line after 20 s were counted. The result is given as the percentage of flies that passed the line. At least 100 flies were analyzed for each genotype and for each time point (1, 15, and 30 days).

### 2.8. Statistics

The software GraphPad Prism was used for statistical analysis, applying the one-way or two-way ANOVA with Bonferroni postdoc test. Error bars represent standard error of the mean (s.e.m.). Asterisks represent * *p* < 0.05, ** *p* < 0.01, *** *p* < 0.001.

## 3. Results

### 3.1. Basic Characterization of the Models

**Figure 1:**
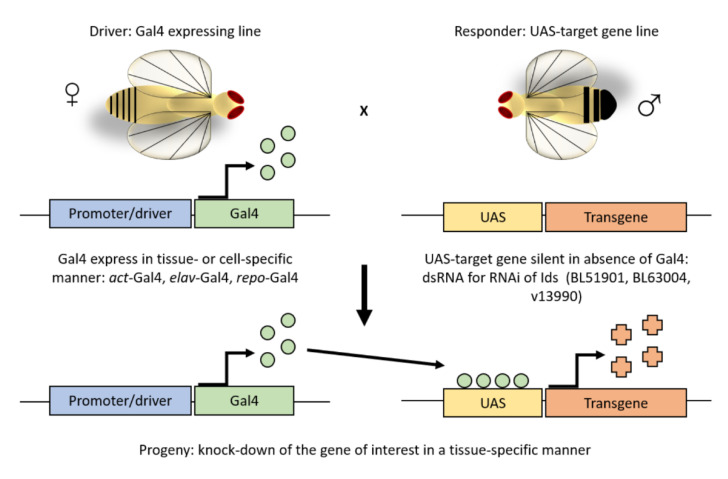
The UAS/Gal4 system. The UAS/GAL4 is the most used method in the fruit fly to ectopically express or knockdown a gene of interest in a tissue- or cell-specific manner. It involves two independent transgenic lines, which are based on the yeast transcriptional activator GAL4 and its high-affinity binding site, the upstream activating sequence (*UAS*). Crossing a tissue-specific GAL4 line to an effector line that carries the *UAS* fused to a gene of interest, progeny with both the GAL4 and *UAS* components express the gene of interest in an activator-specific manner. As in this study, such approach allows for silencing of the *Ids* gene by overexpressing an UAS- *Ids*-RNAi construct.

The UAS/Gal4 system is the fastest method to obtain a *Drosophila melanogaster* knockdown model. It allows downregulating the expression of the gene of interest in a tissue-specific manner. In this study, we crossed three RNAi lines for the *Ids* gene (BL51901, BL63004, v13990) with three different driver lines: *act*-Gal4 for an ubiquitous downregulation of the *Ids* gene, *elav*-Gal4 for pan-neuronal downregulation, and *repo*-Gal4 for downregulation in glial cells (Figure 1). All drivers were also crossed with the wild type line *w^1118^* and these offspring were used as controls.

**Figure 2:**
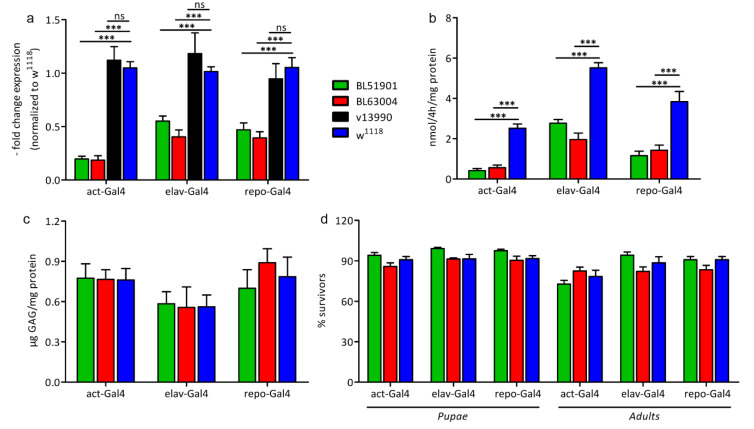
Characterization of the *Ids* knockdown models. (**a**) *Ids* gene expression measured by RT-qPCR and normalized to *w^1118^* (*n* = 25 flies for *act*-Gal4, *n* = 75 heads for *elav*-Gal4 and *repo*-Gal4, all divided in 5 replicates); (**b**) IDS-specific enzymatic activity (*n* = 15 flies for *act*-Gal4, *n* = 45 heads for *elav*-Gal4 and *repo*-Gal4, all divided in 5 replicates); (**c**) GAGs storage (*n* = 60 flies for *act*-Gal4, *n* = 180 heads for *elav*-Gal4 and *repo*-Gal4, all divided in 4 replicates); (**d**) lethality test: for each genotype, L1-L2 larvae were collected on apple juice agar plates and fed with yeast paste; the number of emerging pupae and adults (alive 24 h after hatching) was counted (*n* = 5, in groups of 20–25 larvae for each sample, tot *n* = 100). Data were measured upon ubiquitous (*act*-Gal4), pan-neuronal (*elav*-Gal4) and glial (*repo*-Gal4) knockdown of *Ids*/*CG12014*, with 3 different RNAi fly lines (BL51901, BL63004, v13990) and *w^1118^* wild type control. Due to the negative results obtained with RT-qPCR, v13990 was excluded from subsequent analyses. Data are mean ± s.e.m. *** *p* < 0.001, ns = not significative.

To evaluate the reliability of these models, we first analyzed the capability of these RNAi lines to downregulate the *Ids* expression. The analysis revealed a decrease in mRNA expression by about 80% as compared to the *w^1118^* level (Figure 2a) upon ubiquitous knockdown of *Ids*/*CG12014* with two of the three lines used (BL51901 and BL63004). The expression was also significantly downregulated using a neuronal- or glial-driver line mediated knockdown of *Ids*. In particular, with the neuronal driver *elav*-Gal4 or the glial driver *repo*-Gal4 in combination with the BL51901 RNAi line the *Ids* expression was reduced by about 45% and 53%. Using these driver lines in combination with the BL63004 RNAi line, *Ids* knockdown was determined to be 60% effective in neurons and in glia cells. Expression of the v13990 RNAi line in combination with all three different driver lines resulted in no change of *Ids* mRNA expression. Therefore, this stock was excluded from all subsequent analyses. In sum, the observed knockdown efficiency for the BL51901 and BL63004 *RNAi* lines is in good agreement with previously published data for the *Drosophila* MPS IIIA knockdown model [12].

To assess whether the downregulation of the gene expression corresponded to a decreased IDS protein and consequently to decreased IDS activity, we performed an enzymatic activity assay. As shown in Figure 2b, in all RNAi-dependent knockdowns of *Ids* the enzymatic activity was significantly reduced as compared to *w^1118^* controls: by about 80% in both ubiquitously mediated knockdowns, by 50% (BL510901) and 65% (BL63004) in the pan-neuronal knockdown, and by about 70% in the glia cell driver mediated knockdown. In line with the reduced *Ids* mRNA expression in our RNAi mediated knockdown models, analysis of enzymatic activity revealed a concomitant decrease in IDS enzymatic activity.

Therefore, we further analyzed the glycosaminoglycan storage to reveal the suitability of the *Ids* RNAi knockdown approach in fruit flies as disease model. However, no increase in GAG storage was found compared to controls (Figure 2c). This was unexpected, especially in the ubiquitously mediated knockdown models, where *Ids* is very strongly downregulated. In contrast, in a fly model for MPS IIIA in which a knockdown of N-sulfoglucosamine sulfohydrolase (SGSH) enzyme mRNA was determined to be ~90% effective upon ubiquitous expression, a significant increase in heparan sulfate was already observed in 1-day old flies. However, a direct comparison between the GAG storage and the reduction of enzymatic activity cannot be made, as the GAG detection approaches are different and no data are shown about the enzymatic activity of the MPS IIIA model. [12].

Next, we performed a lethality test to assess whether we can detect any effect of the knockdown approaches on the survival rate. However, as shown in Figure 2d, no differences were detected. In all *Ids* knockdown models, neither the survival from larva to pupa nor the metamorphosis to the adult phase was affected, contrary to the observations in the *Drosophila* MPS VII model [13].

Our data are unexpected; however, the data can be explained by residual gene expression and enzymatic activity (about 20% for both) upon ubiquitous mediated RNAi expression. Indeed, it was already reported that a residual activity of only 1–10% may be sufficient to rescue a pathological phenotype in MPS II [23,24,25].

### 3.2. Molecular Analysis of Pathology Markers

A recent study in a MPS II zebrafish model highlighted that markers of developmental skeletal defects were affected before any evident GAG accumulation [11]. This made us speculate that other pathology markers could be altered at the molecular level before any evident changes in GAG storage.

**Figure 3:**
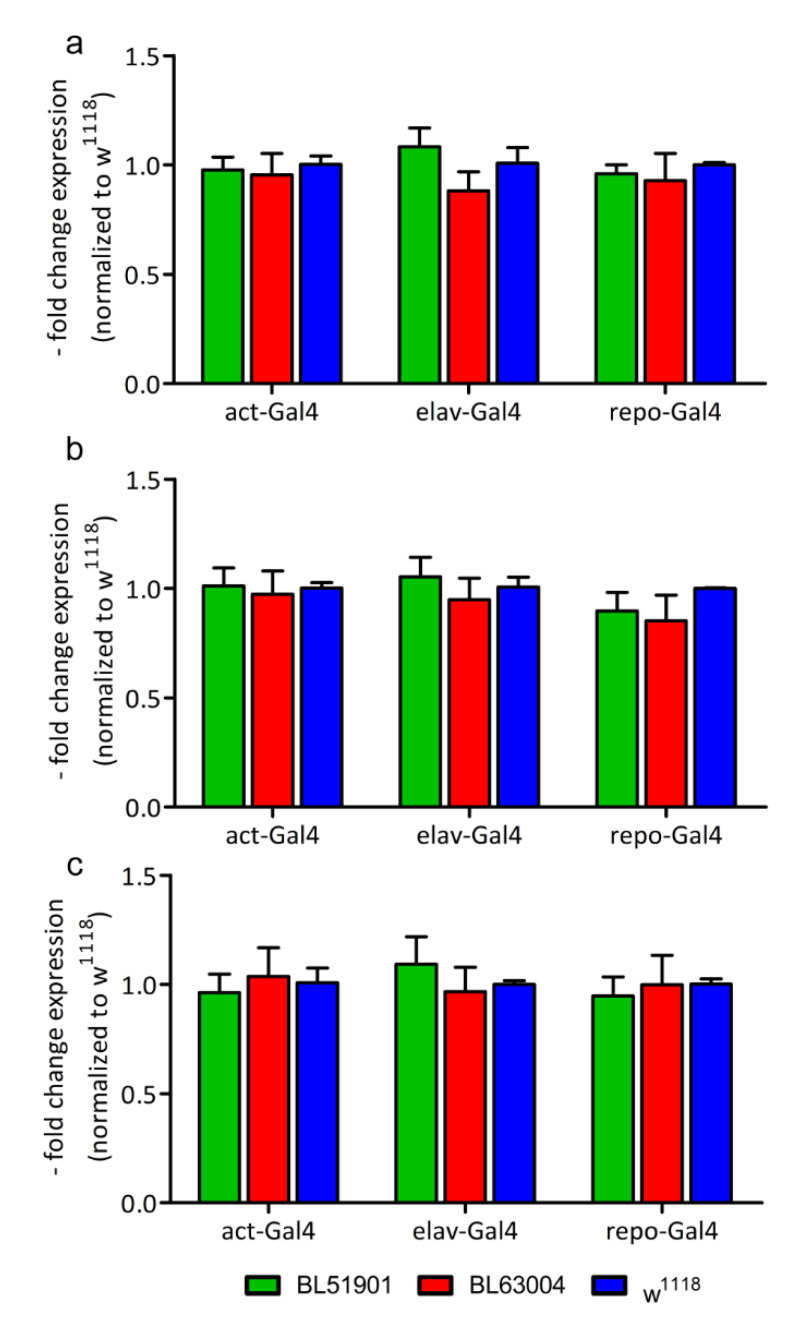
Molecular analysis of pathology markers. Gene expression of (**a**) *Atg8a*, Autophagy-related protein 8a; (**b**) *Lamp1*, Lysosomal-associated membrane protein 1; and (**c**) *Rab11*, Ras-related protein Rab-11, measured by RT-qPCR. RNAi’s data are normalized to *w^1118^* controls. Data were measured upon ubiquitous (*act*-Gal4), pan-neuronal (*elav*-Gal4) and glial (*repo*-Gal4) knockdown of *Ids*/*CG12014*, with 2 different RNAi fly lines (BL51901, BL63004) and *w^1118^* wild type controls. *n* = 25 flies for *act*-Gal4, *n* = 75 heads for *elav*-Gal4 and *repo*-Gal4 (all divided in 5 replicates). Results are mean ± s.e.m.

To assess this hypothesis, we analyzed three genes involved in the endolysosomal pathway well known to be dysregulated in MPS II [2,4,26] by RT-qPCR: Autophagy-related protein 8a (*Atg8a*), which encodes an ubiquitin-like protein and whose product has a role in autophagosome formation; Lysosomal-associated membrane protein 1 (*Lamp1*), a lysosomal marker well known to be overexpressed in MPS II; *Rab11* (Ras-related protein Rab-11, Member RAS Oncogene Family), a small monomeric Ras-like GTPases involved in the regulation of the endomembrane trafficking and the endocytotic recycling. As depicted in Figure 3, RT-qPCR analysis revealed that the transcript levels of none of these genes were changed. Thus, the endolysosomal pathway seems to be unaffected by the downregulation of the *Ids* gene and the concomitant decrease of the enzymatic activity. 

### 3.3. Locomotion Behavioral Studies

As MPS II patients present significant neurological problems and locomotor dysfunctions, behavioral assays are useful methods to study these phenotypes. 

**Figure 4:**
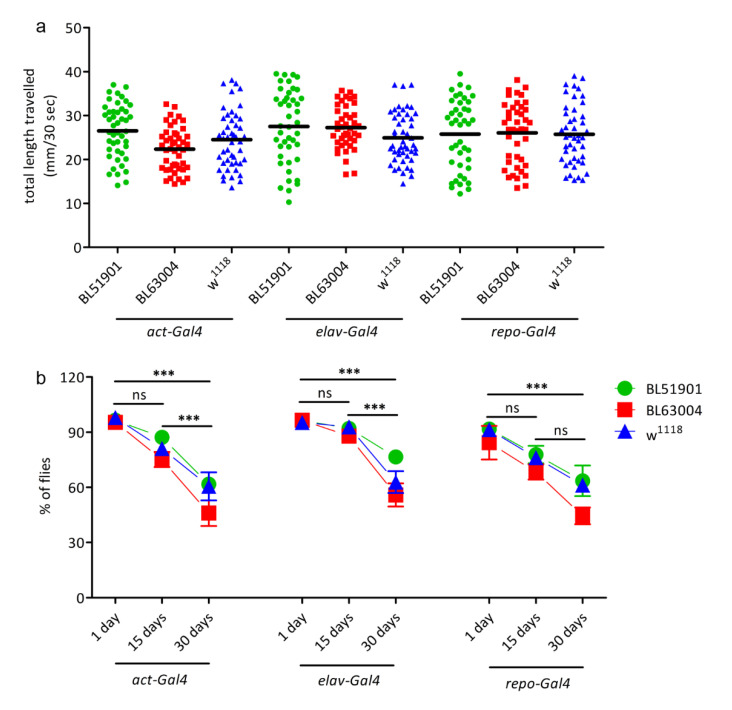
Locomotion behavioural studies. (**a**) Crawling assay: L3 larvae were transferred in groups of 5 on PBS-agarose plates and video-recorded for 1 min. All videos were then analysed using ImageJ to calculate the total distance travelled (in mm) by each larva in 30 s (*n* = 50). (**b**) Climbing assay: negative geotaxis assay to observe motor function decline. Fifteen to twenty adult flies for each genotype were transferred in a 19.5 cm glass tube, gently tapped down to the bottom of the vial and their natural climbing against gravity recorded for 30 s with a camera. The number of flies able to cross a 10 cm line after 20 s was noticed and reported as the percentage against the total number of flies in the tube. The test was repeated 8 times for each group. The assay was repeated at 1, 15, and 30 days after hatching for each group. Significant differences were seen only between different ages, but not in comparison to the *w^1118^* controls. *n* = 100 for each genotype at each age. Data were measured upon ubiquitous (*act*-Gal4), pan-neuronal (*elav*-Gal4), and glial (*repo*-Gal4) *Ids*/*CG12014* knockdown animals, with 2 different RNAi fly lines (BL51901, BL63004) and *w^1118^* wild type controls. Results are mean ± s.e.m., *** *p* < 0.001, ns = not significative.

To finally assess whether *Ids* downregulation resulting in diminished enzymatic activity could lead to behavioral defects in these fruit fly models, crawling and climbing assays were performed. The distance travelled by larvae of any of the knockdown RNAi lines in combination with either ubiquitous, neuronal or glial driver did not differ from *w^1118^* controls (Figure 4a) indicating no locomotor defects in this stage. The climbing assay showed a reduction in the negative geotaxis behavior of the flies with increasing age. However, 15- and 30-day-old flies of all knockdown lines together with all different driver lines showed a similar reduction in the climbing ability with no significant difference in comparison to *w^1118^* controls (Figure 4b). These results suggest no age-dependent neurodegeneration, contrary to what was observed in *Drosophila* models for MPS IIIA and MPS VII, in which climbing defects were seen starting at the age of 1 week and worsening with increasing age [12,13]. In particular, in the MPS IIIA, 45–65% of pan-neuronal *SGSH* knockdown flies are unable to cross the defined cut-off line already at day 1, and 65–85% at day 15 [12], whereas no differences were detected among 1-, 15-, and 30-day-old flies of the MPS II fruit fly models as compared to controls.

Therefore, these data supported the results obtained by GAGs and lethality tests, and they are in line with the hypothesis that residual enzyme activity is still enough to prevent pathological phenotypes.

## 4. Conclusions

In summary, we developed and characterized the first ubiquitous, pan-neuronal, and glial *Drosophila melanogaster* knockdown models for MPS II by using the RNA interference approach. We used three different RNAi lines (BL51901, BL63004, v13990) for the knockdown of the *Ids* gene. Two lines (BL51901, BL63004) showed a significant downregulation upon ubiquitous, neuronal or glial expression.

Despite a strong knockdown of *Ids*/*CG12014* and a reduction of the enzymatic activity, GAG storage, survival, molecular pathology markers (*Atg8a, Lamp1, Rab11*) and behavior were not affected. Our result is probably caused by the residual IDS activity, maintaining a normal phenotype in all tested genotypes. This is in agreement with the fact that, unlike other MPSs, only MPS II patients show never any residual IDS activity [27]. This suggests that the pathological phenotype in these patients appears only when *Ids* gene expression is completely lost. Moreover, it was previously reported that a residual activity of only 1–10% may be sufficient to rescue a pathological phenotype in MPS II [23,24,25].

In sum, this proof-of-principle study revealed that an RNAi mediated knockdown of *Ids* is not sufficiently effective to completely abolish its expression. Thus, it is not suited to establish an MPS II disease model in the fruit fly, contrary to an RNAi mediated knockdown of the SGSH gene, which was successfully applied for generating an MPS IIIA *Drosophila* model showing severe phenotypes on the first day of life [12].

Thus, we provide strong evidence not to use the RNAi knockdown approach for the establishment of an MPS II model in the fruit fly. We rather recommend doing an *Ids* gene knockout by a conventional homologous recombination or CRISPR/Cas9 approach to completely abolish *Ids* expression and to obtain the total absence of enzymatic activity. These MPS II knockout models will show the expected characteristics already in early developmental (e.g., in larval stages or on day 1 of adulthood) and then enable to analyze the neuropathogenesis and other basic and therapeutic studies in stages comparable to the infantile onset of the disease.

## Figures and Tables

**Table 1:** RT-qPCR primers. Fwd, Forward; Rev, reverse; rp49, ribosomal protein 49; *Ids*, iduronate 2-sulfatase; *Atg8a*, Autophagy-related protein 8a; *Lamp1*, Lysosomal-associated membrane protein 1; *Rab11*, Ras-related protein Rab-11.

Gene	Fwd Primer	Rev Primer
*rp49*	GCTAAGCTGTCGCACAAATG	GTTCGATCCGTAACCGATGT
*Ids*	GTTTACAGCCAGCAATCCCT	CGCCAGTAACTGTAGAAATCGT
*Atg8a*	GCAAATATCCAGACCGTGTG	AGGAAGTAGAACTGACCGAC
*Lamp1*	CAACCATATCCGCAACCATCC	GTAAAGTTTCCCTCCCTAGCC
*Rab11*	ATGCGTTTGTCAGCCACAGTT	CCGGATGTTGTTGCTTTCG
